# The Hedgehog Signaling Pathway Emerges as a Pathogenic Target

**DOI:** 10.3390/jdb5040014

**Published:** 2017-11-28

**Authors:** Margery G. Smelkinson

**Affiliations:** Biological Imaging Section, Research Technologies Branch, National Institute of Allergy and Infectious Diseases, National Institutes of Health, Bethesda, MD 20892, USA; msmelkinson@gmail.com; Tel.: +1-240-669-5443

**Keywords:** Hedgehog, Gli, influenza, Epstein Barr virus, Hepatitis B and C, HIV, H. pylori, fibrosis, immunity

## Abstract

The Hedgehog (Hh) signaling pathway plays an essential role in the growth, development, and homeostatis of many tissues in vertebrates and invertebrates. Much of what is known about Hh signaling is in the context of embryonic development and tumor formation. However, a growing body of evidence is emerging indicating that Hh signaling is also involved in postnatal processes such as tissue repair and adult immune responses. To that extent, Hh signaling has also been shown to be a target for some pathogens that presumably utilize the pathway to control the local infected environment. In this review, we discuss what is currently known regarding pathogenic interactions with Hh signaling and speculate on the reasons for this pathway being a target. We also hope to shed light on the possibility of using small molecule modulators of Hh signaling as effective therapies for a wider range of human diseases beyond their current use in a limited number of cancers.

## 1. Introduction: Basics of the Hedgehog Signaling Pathway and Its Evolutionary Conservation

The Hedgehog (Hh) family of ligands are secreted signaling molecules essential for embryogenesis and tissue homeostatis in the adult [1,2,3]. The Hh pathway was originally identified for its developmental role in patterning the *Drosophila* embryo, and Hh pathway components have subsequently been found to be remarkably conserved between invertebrates and vertebrates where the pathway also plays key roles in several types of cancers, some of which are being treated with drugs that inhibit signaling [3,4]. 

**Figure 1:**
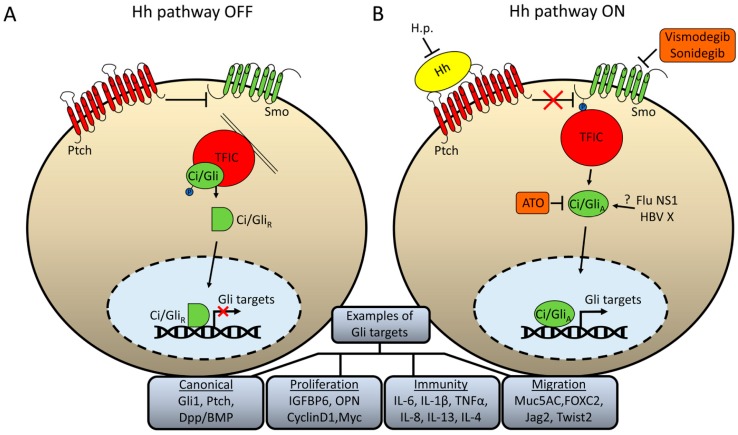
Schematic illustration of Hh signaling and how pathogens may modify pathway activity. (**A**) In the absence of Hh ligand, the receptor Ptch inhibits Smo activation. Ci/Gli is retained in the cytoplasm tethered to microtubules (in flies) or primary cilia (in mammals) through a complex of several proteins, simplistically termed here as “transcription factor inhibitory complex” (TFIC). This complex promotes Ci/Gli phosphorylation, which results in partial proteolysis to a repressor form that can readily enter the nucleus and repress expression of some Gli targets. (**B**) When Hh ligand binds to Ptch, inhibition upon Smo is relieved and the C-terminus of Smo is phosphorylated which promotes the release of Ci/Gli from the TFIC. The activated form of Gli/Ci can enter the nucleus and activate expression of Hh targets. Examples of canonical Gli target genes as well as those involved specifically in proliferation, immunity, and migration are given. Helicobacter Pylori (H.p.) has been proposed to act, in part, by repressing expression of the Hh ligand. Influenza NS1 and HBV X protein have been proposed to interact directly with Gli/Ci, but the precise mechanism by which they affect transcriptional activity has not been fully elucidated. The diagram also shows the pathway components which can be inhibited by FDA-approved small molecules: Vidmodegib and Sonidegib inhibit the activity of Smo, whereas arsenic trioxide (ATO) inhibits the activity of Gli1/2.

The Hh ligand acts as a morphogen to control cellular fates by signaling at narrowly defined durations and concentrations [1,2]. In the absence of Hh, the primary transcriptional effector(s)—Cubitus interruptus (Ci) in Drosophila and Gli-2 and Gli-3 in mammals—are tethered to microtubules in the fly or primary cilia in mammals through a transcription factor inhibitory complex (TFIC) (Figure 1A). This complex contains kinases responsible for phosphorylation and consequent partial degradation of Gli/Ci into transcriptional repressors that lack the C-terminal transactivation domain and nuclear export sequences [1,2,3]. As these shorter protein sequences retain their N-terminal nuclear localization signals, they readily enter the nucleus and inhibit expression of a subset of target genes. 

Cells that express the 12-pass transmembrane protein, Patched (ptc), along with coreceptors can receive the Hh ligand and transduce the signal [1,2]. Hh binding to Ptc initiates a phosphorylation cascade of the G-protein coupled receptor-like, seven-pass transmembrane domain protein Smoothened (Smo) resulting in its surface accumulation and a conformational change that is important for triggering downstream effects (Figure 1B). Although there is some divergence between flies and mammals in steps that relay Smo activation to downstream components [2,5], the culmination in all species is a disruption of the TFIC. This leads to the activation of some Hh target genes simply by inhibiting repressor formation. However, further activation via Fused in the fly [6,7,8] or de-repression by Su(fu) in mammals [9,10,11] results in the conversion of Gli/Ci into a labile transcription factor that can activate the full range of targets.

One of the key differences between flies and mammals is the redundancy in pathway components [2]. Whereas Drosophila has only one Hh ligand and one primary receptor, mammals have three ligands (Sonic Hedgehog-Shh, Indian Hedgehog-Ihh, and Desert Hedgehog-Dhh) and two primary receptors (Ptch1 and Ptch2). Similarly, Drosophila has only one transcriptional effector, Ci, which can act as both an activator and repressor, whereas mammals have three, Gli1, Gli2, and Gli3, where Gli2 is the primary activator, Gli3 is the primary repressor, and Gli1 is a target gene that acts as an activator in a positive feedback loop [12]. There are also differences in how the message is relayed from Smo to downstream components. Namely, mammalian cells require intracellular transport components housed in primary cilia [13]; this subcellular compartmentalization is not required in Drosophila [5]. Regardless of these differences, the method by which canonical Hh signals as a morphogen in all systems is understood to be by creating precise balances between repressor and activator forms of Gli/Ci, which activate target genes with varying numbers and affinities of transcription factor binding sites [14,15].

Non-canonical pathways have also been described more recently which are independent of Gli activity or independent of Hh activation, suggesting that the influence of the Hh pathway on cellular processes may be far more expansive than originally thought [16]. If this is the case, presumably regulating this pathway provides a broad target to pathogens, such as viruses, that have a limited number of genes and effector avenues for controlling the host environment. 

## 2. Hh Signaling as a Target of Pathogens

While much is known about Hh signaling in embryogenesis [12,17], it has been shown, more recently, that signaling also occurs postnatally in several tissue types such as the skin, lung, gut, and within the haemopoatic and immune systems [18,19,20,21,22,23,24,25]. A clear role has been established for this postnatal signaling in maintaining tissue homeostasis, stem cell maintenance, and regulating haematopoiesis and lymphopoiesis [25,26,27,28,29,30]. However, more recently, it has also been shown that Hh signaling is activated in response to encounters with pathogens, wounding, and/or damaging agents. As the body of evidence supporting Hh signaling in these latter processes grows, perhaps unsurprisingly, so does the data demonstrating the Hh pathway as a target of several pathogens. Examples of Hh-interacting pathogens are discussed below.

Hepatitis B and C (HBV and HCV, respectively) are major causes of liver cirrhosis and hepatocellular carcinoma (HCC) worldwide [31]. Initial findings by Pereira et al. showed that livers from patients with chronic HBV and HCV infection displayed an increase in hepatocyte production of Hh ligands and an accumulation of Hh-responsive cells with higher levels of pathway activity correlating to more dire outcomes [32]. Complementary studies further confirmed that treatment of liver cells in vitro with the whole HBV replicon or with serum from HCV-infected patients increased expression of Hh targets in a Gli-dependent manner [33,34] and led to pro-fibrotic effects [33]. In the case of HBV, the viral protein causing this effect was revealed to be HBV X (Figure 1B) which increases Gli1 protein stability and promotes nuclear accumulation, an interaction shown likely to be direct through a series of in vitro binding assays [34,35,36]. While the precise motive by which these viral activities promote infections remains unclear, Choi et al. showed that increasing Hh signaling in liver cells promoted permissiveness for HCV replication, implying the presence of a positive feedback loop between pathway activation and viral production [37].

Hh target genes are also increased in Epstein–Barr virus (EBV)-derived nasopharyngeal carcinoma (NPC) tissue, NPC-derived cell lines, and in EBV infected epithelial cells, in vitro [38]. Further mechanistic studies revealed that the EBV-dependent increase in Gli1 expression correlated to a decrease in expression of Human Leukocyte Antigen, which is involved in presentation of viral antigens to cytotoxic T cells, which may limit the recognition of EBV by the immune system [39]. Hh activity is also upregulated in mice kidneys afflicted with HIV-associated nephropathy and in a human podocyte cell line infected with HIV [40]. This increase was associated with enhanced expression of proliferation and migration markers, loss of kidney filtration barrier function, and increased permeability, which could presumably augment viral dissemination and decrease host defenses, among other effects. 

Helicobacter Pylori (H.p.) is a gram-negative bacterium found in the stomach and is the major cause of chronic atrophic gastritis and gastric cancer worldwide [41,42]. During the early stages of H.p. infection, Hh was shown to be upregulated in HCl-secreting parietal cells in an in vivo mouse infection model and in epithelial cell cultures grown in vitro from whole dissociated gastric glands [43,44,45]. This upregulation of signaling appeared to be due, in part, to the inflammation/repair response as this promoted macrophage recruitment to the infected area and was dependent upon NFkB induction. Prolonged exposure of H.p., however, resulted in a decrease of Hh expression in the gastric epithelium and an associated loss of parietal cells in the gastric gland in Mongolian gerbils, an established animal model for H. pylori infection, and in patients with H.p.-dependent chronic gastritis analyzed postmortem [46,47]. Eradication of H.p. infection could often restore Hh expression and frequently reversed tumor transformations [48,49,50,51]. This decrease of Hh signaling over the course of H.p. infection is partially in response to macrophage secretion of regulatory cytokines [43]. However, it has been also been proposed that H.p.-dependent upregulation of caudal-type homeobox 2 (CXD2), a protein that can bind directly to the promotor of Shh, is responsible for repressing expression, suggesting that this may be an evolved mechanism for regulating Hh pathway activity [52,53,54]. 

More recently, we reported that the Hh signaling pathway is also a direct target of the influenza A virus, linking pathway activation to the pathology of the virus [55]. This observation was originally made using *Drosophila melanogaster* as a model organism to screen for cell non-autonomous activities of disease genes and also an organism where Hh signaling has been shown recently to be linked to innate immunity [56,57,58,59,60]. 

**Figure 2:**
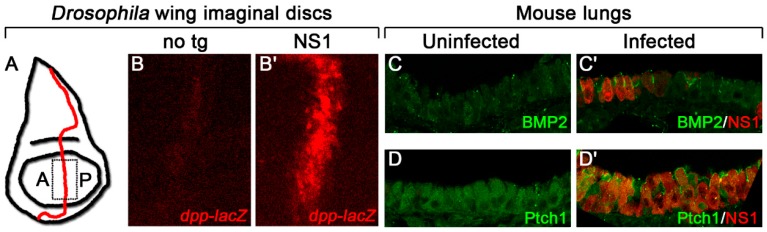
Influenza NS1 increases Hh target gene expression in *Drosophila* and mice. (**A**) Schematic diagram of a *Drosophila* wing imaginal disc with the anterior/posterior (A/P) border, the domain where Hh signaling is active, demarcated in red. The dashed box represents the area of the disc that was imaged in B. (**B**) Influenza NS1, expressed from a transgene in the wing imaginal disc, increases expression of the Hh target gene reporter, *dpp-lacZ*, at the A/P border compared with a disc with no transgene (no tg). (**C**,**D**) Infected mouse lungs show a cell-autonomous increase in expression of the Hh targets, BMP2 (**C**) and Ptch1 (**D**) compared with uninfected lungs. Target proteins are in green, NS1 is in red.

In larval wing precursors (Figure 2A) expressing the influenza gene Nonstructural protein 1 (NS1), we detected a dramatic increase in expression of the Hh target gene, *decapentaplegic* (*dpp*) (Figure 2B), as well as the Dpp target, pMad, and a corresponding increase in distance between the adult wing veins L3 and L4, phenotypes indicative of increased Hh signaling [55,61,62]. Expression of BMP2, the mammalian homologue of *dpp*, and the Hh target, Ptch1, was also enhanced cell-autonomously in infected mouse lungs, indicating that the effect of NS1 on Hh signaling is conserved between species (Figure 2C,D) [55]. As NS1 is one of 14 proteins encoded by the influenza A virus whose main role is as a virulence factor to interact with host proteins to promote viral growth and maturation, it is perhaps expected that this protein may have evolved to exploit a conserved signaling pathway [63,64,65]. 

Using *Drosophila*, a forward genetic screen was performed to identify point mutations that might disrupt the Hh-dependent activity of NS1. A point mutation was recovered resulting in an alanine to valine substitution at position 122 that reduced this activity in flies and in transfected cells [55]. Interestingly, however, when the A122V mutation was incorporated into a mouse-adapted influenza A virus, it cell-autonomously enhanced expression of some Hh targets in the mouse lung and significantly hastened lethality. These results indicate that, in addition to its multiple intracellular functions, NS1 plays a vital role in activating—but at the same time, mitigating—the activity of a highly conserved signaling pathway during infection. Interestingly, no mutation at position 122 of NS1 has been identified previously in any influenza strain, which may reflect the critical role of NS1 in restraining signaling in order to protect the host. This muting of Hh signaling may be utilized to dampen deleterious effects that could be caused by Hh signaling (discussed below) to ensure optimal viral maturation prior to dissemination. 

The precise mechanism by which Hepatitis viruses, HIV, EBV, and H.p. interact with Hh signaling has not been completely elucidated, however, all interactions and often the deleterious effects could be blocked by eliminating Gli [34,39,66] and/or by blocking Hh signaling with potent inhibitors [33,35,38,40]. Similarly, in flies, NS1-dependent pathway activation was blocked by over-expressing the receptor Ptc (which recruits Hh ligand and prevents it from dissipating to other cells), an RNA interference (RNAi) molecule directed to Ci, or other factors with inhibitory roles in signal transduction [55]. Likewise, constitutively activating signaling using a phosphomimetic form of Smoothened promoted activity. Thus, similar to other viruses, influenza NS1 requires pathway activation to upregulate Hh target genes. 

The mechanism by which NS1 modulates Hh signaling was analyzed using the extensive array of genetic and molecular tools available when using *Drosophila* as a model organism to study disease genes. For example, a series of genetic epistasis experiments and quantitative imaging experiments using Förster resonance energy transfer-Fluorecence lifetime imaging (FRET-FLIM) assays revealed that NS1 interacts directly with the transcriptional mediator, Ci/Gli1, parroting the HBV X/Gli1 interaction [36,55]. Furthermore, this analysis showed that the A122V mutation significantly impeded this interaction. Importantly, pathway activity was not required simply to stabilize the full-length form of Ci and eliminate repressor formation, but additional pathway components with positive roles in signaling were also required for NS1 activity. Taken together, these data suggest that viral proteins likely do not activate the Hh pathway or expression of target genes themselves directly, but rather that Hh signaling is first activated by canonical signaling and then a viral interaction intervenes to alter the readout of the pathway activity. How Hh signaling is initially activated during infection remains an intriguing question. 

## 3. Why Might Hh Signaling Be a Frequent Target for Pathogens?

To ensure efficient use of their genomes, pathogenic interactions are usually established with more centrally connected host proteins so that several processes may be targeted concomitantly. These virulence factors constantly evolve their interface residues, either to evade or to optimize their binding capabilities to host proteins [67]. Thus, some viruses may have evolved the ability to modulate Hh signaling due to its substantial involvement in several processes such as wound healing and the role it plays in immunity. 

### 3.1. Hh Signaling Modulates Wound Repair and Tissue Fibrosis

**Figure 3:**
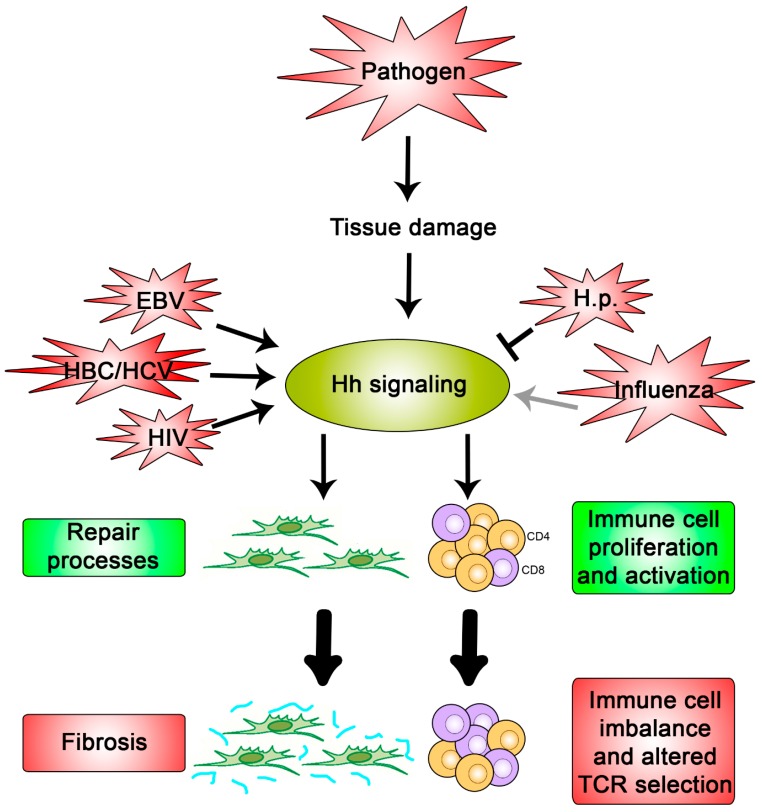
Hypothesis for what occurs during a pathogenic interaction with Hh signaling. Pathogens that cause damage to the host tissue promote activation of Hh signaling due to the key roles it plays in repair processes and immunity (green boxes). If not regulated properly, these cellular processes can cause fibrosis and an imbalanced immune response, respectively (red boxes). Pathogens, such as Influenza, EBV, HBC, HCV, HIV, and H.p., have been shown to directly modulate pathway activity once signaling is activated and may do so in order to exacerbate or restrain these detrimental outcomes.

As Hh signaling has significant roles in tissue homeostasis and remodeling, we speculated that Hh signaling may be activated as a part of a defense mechanism during infection to promote tissue repair (Figure 3). Indeed, a firm role for Hh signaling in wound repair has been established in several cells types such as the lung, skin, and pancreas where damaged areas correlate with Hh activation. 

In the lung, acute activation of Shh signaling in epithelial cells was observed upon naphthalene injury, which was strikingly similar to the activation observed during prenatal development [68]. Shh is also upregulated in lung homogenates during hyperoxia-induced injury and recovery [69] and bleomycin administration [70], as well as around damaged airways in lungs chronically exposed to fluorescein isothiocyanate (FITC) [22,71]. 

In the skin, Asai et al. found that Ptch1 expression is upregulated in wounds in mice and that *in vitro* stimulation of cultured skin cells with Hh ligand promoted production of angiogenic signals, increased proliferation of fibroblasts, and increased migration, adhesion, and tube formation of endothelial progenitor cells to aid in wound closure [72]. Furthermore, topical gene therapy treatment on wounds using DNA encoding Shh accelerated wound recovery, while commensurate studies showed that wound closure, vascularization, and proliferation were inhibited at wound sites by intraperitoneally injected cylopamine, a potent Smoothened antagonist [21,72,73,74]. 

Similarly, Shh signaling in pancreatic fibroblasts is required for tissue repair during the onset of pancreatic cancer and pancreatitis due to a mutation in the proto-oncogene, K-ras, as a deletion of one allele of Gli1 or mice lacking expression of Shh in the pancreatic epithelium significantly impaired tissue remodeling and cytokine expression in this model [75]. This study also confirmed the presence of Ptch1 on the surface of several immune components including peripheral T cells, suggesting that Hh ligand secreted from the damaged areas can activate the local immune network to work simultaneously to repair the tissue. Interestingly, Gli1 also plays a key role in pancreatic tumorigenesis by enhancing expression and activation of Signal transducer and activator of transcription 3 (STAT3) by direct and Hh-dependent upregulation of IL-6 expression [76]. Therefore, a positive feedback loop exists whereby Hh signaling helps initiates tumor formation which, in turn, triggers Hh signaling to assist in tissue repair. 

Where repair processes fall short, however, tissue fibrosis can occur, marked by the formation of detrimental scar tissue. Perhaps not surprisingly, there is also a strong link between tissue fibrosis and Hh signaling in several tissues inflicted with chronic ailments, primarily driven by the Hh-dependent activation of epithelial to mesenchymal transition of cells and the resultant excessive extracellular matrix deposition [77]. Pulmonary fibrotic diseases such as idiopathic pulmonary fibrosis (IPF) [78,79], interstitial lung disease [80,81,82], and usual and nonspecific interstitial pneumonia [83] are all characterized by expression of Shh and Ptch1 in areas of fibrosis. Additionally, Gli1nlacZ/+ mice show an increased number of Hh-responsive cells in bleomycin-induced fibrosis where overexpression of Shh can further augment damage [84]. 

Hh ligand secreted by liver cells in response to damage causes cellular differentiation of fibroblasts and myofibroblasts which are critical for normal liver regeneration [37,85,86]. However, similarly to the lung, excessive signaling leads to fibrosis, which can cause cirrhosis and HCC if not controlled [37,87]. Syn et al. demonstrated that Hh upregulates expression of osteopontin (OPN), a cytokine involved in wound healing, in a Nonalcoholic Steathohepatitis-related liver fibrosis, where a reduction in either Hh signaling or OPN reduced fibrogenesis [88]. This was postulated to be through a direct interaction between Gli and Gli-binding sites in the OPN promotor [89]. Moreover, HBV and HCV infections are leading causes of liver fibrosis which is likely mediated through Hh-signaling enhancement (the role of Hh signaling in HBV and HCV pathogenesis is discussed above) [32,33,35]. 

Hh ligand and target genes, including those involved in extracellular matrix (ECM) production, have also been shown to be upregulated in a mouse model of renal fibrosis following injury where protection was provided by cyclopamine treatment or by deficiency in Gli1 [90,91]. Similarly, transgenic zebrafish over-expressing Hh ligands developed pancreatic fibrosis, and pathway components are found upregulated in patients suffering from pancreatitis [92,93]. Correspondingly, Hh-mediated expression of ECM genes were also upregulated in cultured renal cells and in culture-activated pancreatic stellate, both of which showed augmented proliferation and/or migration [91,94]. 

These data clearly establish the role of Hh signaling in tissue repair processes where unstrained signaling can cause harmful fibrotic outcomes. This aligns with a model in which pathogen-dependent tissue damage causes a Hh-mediated repair response (Figure 3). Some viruses may augment this already active signal to promote irreparable fibrotic damage to ensure viral spread. However, Hh activation at sub-maximal capacity, such as what appears to occur during influenza and in late-stage H.p. infection, may limit formation of fibrotic tissue which may also benefit the pathogen by increasing the time allotted for replication, maturation, and/or dissemination while, at the same time, also maintaining an ample pool of viable hosts available for reinfection. 

### 3.2. Hh Signaling Involved in Immune Pathways and Immune Diseases

The Hh pathway also has postnatal roles in defining the immune response which is conserved from flies to humans, although the relevant signaling output and how it is applied as a defense mechanism differs between vertebrates and invertebrates. In a study conducted in *Drosophila* by Lee et al., Hedgehog signaling was found to be required as a first line of defense against harmful uracil-secreting pathogens in the gut by triggering the production of microbicidal reactive oxygen species (ROS) [60,95,96]. Flies expressing RNAis directed to several canonical Hh pathway components showed reduced ROS production upon bacterial infection and a consequent higher mortality rate. Exogenous expression of the Hh target gene, *Cadherin 99C (Cad99C)*, however, could rescue this phenotype, and subsequent experiments determined that Cad99C-dependent formation of signaling endosomes stimulated the enzyme, dual oxidase (DUOX), to produce ROS in response to uracil detection.

In mammals, Hh signaling takes a multipronged approach in regulating the immune system by controlling several T-cell features such as differentiation, proliferation, and activity [97,98]. Shh ligands, secreted from thymic epithelial cells to T-cell progenitors, influence cell lineage and proliferation [97]. Pro-thymocytes undergoing a series of differentiation stages ultimately mature into either helper or cytotoxic T cells defined by the expression of the cell-surface markers, CD4 or CD8, respectively [99]. During this process, thymocytes progress from double-negative (DN) to double-positive (DP) expressing markers, with several intermediate steps in between classified by CD25 and CD44 expression profiles, then on to mature single-positive cells that can then exit the thymus to the bloodstream [100]. Throughout this process, T-cell receptor maturation and selection is occurring via gene rearrangement such that a diverse array of receptors are produced that can recognize foreign peptides [101]. 

Analysis of thymocyte phenotypes in mouse mutants of Hh pathway components show that Hh signaling is important for regulating T-cell proliferation rates, determining the final differentiation step between CD4 vs. CD8, controlling various steps throughout the conversion process of double negative cells to single positive cell, as well as modulating the strength of T-cell receptor (TCR) signaling to influence TCR repertoire selection [27,28,29,102,103]. Additionally, Ihh expression directly in developing DP T-cells themselves is critical for negatively regulating proliferation and differentiation of thymocytes at earlier stages in the developmental process [104]. Ihh is also expressed directly in mature CD8 T-cells and, through an autocrine manner of signaling, is involved in regulating activity by controlling immunological synapse formation and mediating target cell lysis [98]. Thus, both positive [27,80,81,98,103,104] and negative [28,29,104] roles for Hh signaling have been described for these processes, suggesting that there may be a narrow range of signaling that is optimal for proper T-cell maturation andactivity and for generating proportional representation of subpopulations. 

Direct induction of cytokine expression—secreted factors of immune cells that have an effect on other cells—is another capability of Hh signaling. Peripheral CD4 T-cells retain expression of Hh pathway components and can respond to Hh signaling following TCR-activation [80,81]. Hh signaling can induce clonal expansion of this cell population by enhancing expression of cytokines, such as IL-2, IL-10, and IFNγ, which promote entry into the S-G2 phase of the cell cycle. Additionally, human macrophages stimulated in vitro with recombinant Shh respond by upregulating expression of some cytokines and chemokines, such as IL-6, IL-8, MCP-1, and IL-10, whereas expression of others was reduced in a conditional Hh KO H.p. infection model [105,106]. Shh also induces expression of the pro-fibrotic cytokines, IL-13 and IL-4, in natural killer T-cells during liver fibrosis [107,108,109].

Consistent with Hh controlling immune cell proliferation and cytokine induction, some auto-immune disorders [110,111,112,113] and leukemias [114,115] have been associated with aberrant pathway activation. Allergic asthma is an example of an autoimmune condition where the pathophysiology of the disease is connected to an erroneous immune reaction to aeroallergen inhalation [116]. This disease has been directly linked to Shh production in a murine model where pathway activation results in an over-abundance of conversion of naïve T cells to T_helper_ 2 (Th2) cells [110]. This occurs through a Hh-dependent upregulation of several target genes that specify Th2 cells’ fates such as the cytokines, IL-4 and IL-1rl1 [30,110]. Thus, while Th2 cells are important for protection against extracellular parasites and tissue remodeling upon damage, they are also involved in the pathogenesis of some allergic and inflammatory diseases [117]. 

Similarly, influenza infection strongly induced expression of cytokines CXCL-10 and IL6, the latter at least partially by a direct, cell-autonomous interaction between Hh signaling and NS1 [55]. Interestingly, IL6 (along with other Hh target genes discussed above) was present at comparatively higher levels in animals infected with the more pathogenic virus carrying the A122V point mutation in NS1, thus we speculate that the hastened lethality caused by the mutant virus may be due, in part, to a Hh-dependent over-production of cytokines (often called cytokine storms), which have been thought to be the cause of past influenza pandemics [118].

Collectively, these studies indicate that select populations of immune cells are primed to respond to the Hh signal, often resulting in proliferation and an upregulation of a subset of cytokines. Increasing Hh signaling, as occurs in certain tissues during HBV, HCV, EBV, and HIV infection, may help the virus evade the immune response by disrupting the balance of immune components available to extract the infection (Figure 3). In contrast, limiting full pathway activation, as occurs during influenza infection, may suppress the immune response to evade it and/or protect the host from detrimental outcomes, such as cytokine storms [75,76,97]. 

## 4. Therapeutic Strategies

Small molecule modulators of Hh signaling have been used in basic research for several years now to detect links between signaling and specific phenotypes of interest. Currently, CDC-0449 (Vismodegib) and LDE225 (Sonidegib), both Smoothened inhibitors, and arsenic trioxide (ATO), a Gli1/2 inhibitor, have been approved by the Food and Drug Administration (FDA) to treat basal cell carcinoma (BCC) and certain leukemias, respectively, whereas many others are still in clinical trials (Figure 1B) [3,119,120]. The idea that these approved and yet-to-be-approved molecules might be repurposed to have therapeutic value in humans beyond certain types of cancers is certainly worth exploring [24].

Treatment both in vivo and in vitro with Vismodegib or the Gli inhibitors, Gant-58 and Gant-61, has successfully reversed the detrimental phenotypes caused by several of the ailments mentioned in this review. For example, Vismodegib reduced liver fibrosis and tumor formation in a mouse model of fibrosis-associated HCC [121] and ameliorated early liver fibrosis in a rat model of common bile duct ligation [122]. Vismodegib also reduced the growth of HBV X-expressing tumor xenografts in nude mice and HCC formation in transgenic mice expressing the HBV X protein [35]. Similarly, Gli inhibitors reduced the pro-fibrotic effects and autophagy inhibition in HCV-exposed fibroblasts [33], reduced tumor-sphere formation in several EBV-infected cell lines [38], and decreased the proliferation of Human Papilloma Virus-derived cervical cancer cells [123]. Likewise, ATO was shown to inhibit tumor growth in several types of cancers in both in vitro and in vivo models [124,125,126,127,128,129].

Thus, it stands to reason that therapeutic uses of FDA-approved molecules that inhibit Hh signaling may be expanded for use as potent inhibitors to treat several pathogenic infections. In contrast to the currently available therapies, such as vaccines and antivirals, which target strain-specific and rapidly-mutating viral proteins, treatments that target highly-conserved host targets may ultimately provide superior and continual protection across a broader spectrum of strains.

Interestingly, HIV-infected cells and tissues appear to be responsive to treatment with both Hh antagonists and agonists—the former directed to infected cells, the latter directed to the local uninfected environment. In the former case, Gli inhibitors were shown to decrease HIV-dependent proliferation and migration of mouse kidney podocytes in vitro [40]. In the latter case, treatment of humanized mice with a Smoothened Agonist reduced leukocyte infiltration into the brain by fortifying the blood–brain barrier, thus limiting the viral niche [130,131]. These differences illustrate a circumstance in which the location of the infection (kidney vs brain) can be controlled through contrasting Hh-modulating mechanisms. 

These HIV studies reinforce the importance of carefully selecting a relevant treatment since the cellular processes regulated by this signaling pathway are expansive and unforeseeably complex. This may require a combinatorial approach in which several drugs are targeted to different Hh-dependent processes, which may further strengthen or reduce signaling in a context-dependent fashion. This may be possible by using a combination of drugs that target pathway components with both positive and negative roles in signaling. Additionally, defining the precise mechanism in which viral factors interact with the pathway would, no doubt, restrict the search for useful drugs. 

## 5. Summary

The Hh signaling pathway has emerged as a target of several pathogens in recent years, where cases of both pathogen-dependent increases and decreases in pathway activity have been observed. These changes in signaling correlate to an exacerbated detriment to the host and sometimes also as protection, as in the case of influenza. We speculate that the key postnatal roles that Hh signaling plays in wound repair and the immune response are critical features that would be appealing for pathogenic control. Potential new therapies involving Hh inhibitory and stimulatory compounds that disrupt or reverse these interactions could derive from these findings and suggest an important new avenue for further investigation.
